# Bridging Gaps in Migrant Healthcare: CUAMM’s Experience from 13,103 Visits in Southern Italy

**DOI:** 10.5334/aogh.4666

**Published:** 2025-03-28

**Authors:** Cesare De Virgilio Suglia, Renato Laforgia, Marcella Schiavone, Anna Belfiore, Nicole Laforgia, Annalisa Saracino, Giovanni Putoto, Francesco Di Gennaro

**Affiliations:** 1 Doctors with Africa CUAMM, Bari, Italy; 2 Internal Medicine “A. Murri,” Department of Precision and Regenerative Medicine and Ionian Area (DiMePRe‑J), Polyclinic Hospital, Bari, Italy; 3 Clinic of Infectious Diseases, Department of Precision and Regenerative Medicine and Ionian Area (DiMePRe‑J), University of Bari ‘A. Moro’, Polyclinic Hospital, Bari, Italy; 4 Operational Research Unit, Doctors with Africa CUAMM, Padua, Italy

**Keywords:** Migrant health, healthcare access, social determinant, vulnerability, mobile clinics, labor exploitation, social inclusion, Doctors with Africa CUAMM, health disparities, primary health care, infectious disease, health policy

## Abstract

*Background:* Agricultural migrant workers in rural Puglia, Italy, endure harsh living and working conditions that significantly affect their health and limit access to healthcare. This study evaluates their health status, explores systemic barriers to care, and evaluates the effectiveness of a mobile clinic model, identifying structural obstacles to healthcare access.

*Methods:* Data were collected from 13,103 medical visits conducted between 2017 and 2023 by Doctors with Africa University College for Aspiring Missionary Doctors (CUAMM)’s mobile clinics operating in 12 informal settlements. Demographic, clinical, and socio‑health data from 2,537 unique patients were analyzed. Statistical methods, including multivariate regression, were employed to identify health trends and predictors of healthcare utilization.

*Results:* The patient cohort was predominantly male (95.8%) and aged 19–45 years (83%). Work‑related musculoskeletal disorders were the most common diagnoses (27.3%), followed by respiratory infections (14.3%), dermatological conditions (12.1%), and dental problems (7.2%). Only 18% of patients had a residence permit, and 7% were registered with a general practitioner. Despite significant barriers, the average number of follow‑up visits per patient was 5.6, indicating trust in the mobile clinic model. Barriers included linguistic and cultural challenges, low health literacy, and irregular legal status. Mobile clinics provided not only primary medical care but also referrals and socio‑health guidance, effectively bridging healthcare gaps for this population.

*Conclusions:* This study underscores the health vulnerabilities of migrant workers and the critical role of mobile clinics in addressing their needs. Integrating flexible care models with traditional systems, addressing labor exploitation, and improving living conditions are imperative. Collaborative efforts involving institutions, nongovernmental organizations (NGOs), and academia are essential to ensuring equitable, accessible, and sustainable healthcare for this marginalized population—**leaving no one behind**.

## Introduction

In Italy, the agricultural sector employs about 500,000 migrant workers, nearly half of its workforce. At least 180,000 face exploitation, often living in makeshift settlements or “ghettos” far from urban centers [1, 2]. In Puglia, one of the country’s major agricultural regions, over 60% live in unsafe, rundown structures lacking essential utilities such as water, electricity, and sanitation [3–5]. These conditions, together with systemic exploitation by illegal labor intermediaries (*caporali*), exacerbate their vulnerability [6, 7]. Despite Italy’s universal healthcare system, many migrants encounter linguistic, cultural, and bureaucratic barriers, as well as irregular legal status [1, 3, 4, 7]. Most migrants are subject to strong seasonal migration, moving according to harvest periods, and this negatively affects the continuity of care. Since 2017, Doctors with Africa University College for Aspiring Missionary Doctors (CUAMM) has addressed these issues through its “Supreme” project. By deploying mobile clinics across 12 informal settlements, CUAMM provides free healthcare and social‑health orientation to some of the region’s most marginalized populations. Recognized by the World Health Organization (WHO) as a best practice in migrant health, this model is often the only access point for care, fostering trust and bridging critical gaps [8, 9].

This study investigates the health needs of migrant agricultural workers in Puglia, assesses the effectiveness of mobile clinics, and identifies systemic barriers to accessing care. It focuses on common conditions (musculoskeletal disorders, respiratory infections, and dermatological issues), considers follow‑up visits as an indicator of trust, and examines the role of social factors such as housing, legal status, and access to primary care. The findings offer actionable insights to guide targeted interventions, enhance healthcare delivery, and advocate for policy changes to ensure equitable, continuous care for this vulnerable population.

## Methods

**Figure 1:**
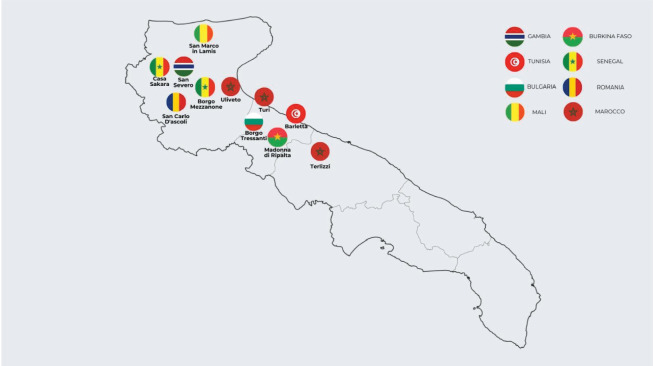
Map of the informal settlements supported by Doctors with Africa CUAMM.

This study analyzed healthcare visits conducted between 2017 and 2023 by volunteers from Doctors with Africa CUAMM in 12 informal settlements inhabited by foreign agricultural workers in the Puglia countryside, as showed in Figure 1. Visits from 2024 were excluded owing to ongoing data collection and continuous updates to the database.

The informal settlements included in this study share common structural and socioeconomic characteristics. They are geographically isolated, often located far from urban centers, with no direct access to essential services such as electricity, running water, and sanitation. Housing conditions are precarious, with only a few permanent structures in masonry; the majority of dwellings consist of makeshift shelters built from sheet metal and other temporary materials.

The medical visits were carried out aboard a mobile clinic staffed by a multidisciplinary team comprising a doctor, nurse, medical resident (specializing in infectious diseases, surgery, dermatology, or other fields), cultural mediator, and social care worker. The caravan was equipped with a medical bed, a large supply of medicines for the most common pathologies, a sphygmomanometer, a pulse oximeter, a portable ultrasound, an electrocardiogram (ECG) device, a glucometer, and rapid tests for human immunodeficiency virus (HIV), severe acute respiratory syndrome coronavirus 2 (SARS CoV‑2), and hepatitis C virus (HCV). On average, 8 days of activity with a camper were carried out each month, with oscillations between 4‑ and 12‑monthly outreach, in relation to the presence of agricultural workers and the possibilities of the nongovernmental organization (NGO). Data collected during these visits—including demographic, socio‑health, anamnesis, and clinical information—were systematically recorded in a Microsoft Access database as well as in an anonymized Excel file. Descriptive analyses were performed to characterize patients at their first recorded visit. Categorical variables were summarized as absolute and percentage frequencies, while continuous variables, such as age, were described using the median, mean, and standard deviation (SD).

Subsequent analyses examined repeated visits, focusing on the frequency of visits (absolute and relative), gender distribution, diagnosed pathologies, and administered therapies.

Factors associated with voluntary follow‑up visits, used as a proxy for trust in the healthcare model, were investigated using simple and multiple logistic regression analyses. Variables were selected through a stepwise bidirectional approach to minimize the Akaike Information Criterion (AIC). Key variables, such as age at the first visit (categorized into ranges) and gender, were included in the model regardless of statistical significance to control for potential confounders. Odds ratios (ORs) and their 95% confidence intervals (CI) were calculated using Blaker’s approach.

Follow‑up visits were considered an important outcome variable, as mobile clinics often represent the only access to healthcare for this population owing to their geographic isolation and multiple structural barriers. Given the lack of alternative healthcare options, patients were encouraged to return for continued care and monitoring, particularly for chronic conditions, treatment adherence, and unresolved medical issues. The length of follow‑up varied on the basis of individual health needs, with some patients requiring multiple visits over several months or years, while others returned only for acute care episodes.

The study was conducted in accordance with the Declaration of Helsinki and national and institutional standards. All statistical analyses were conducted using the R programming language within the RStudio Integrated Development Environment (IDE).

## Results

### Demographic and settlement characteristics

**Figure 2:**
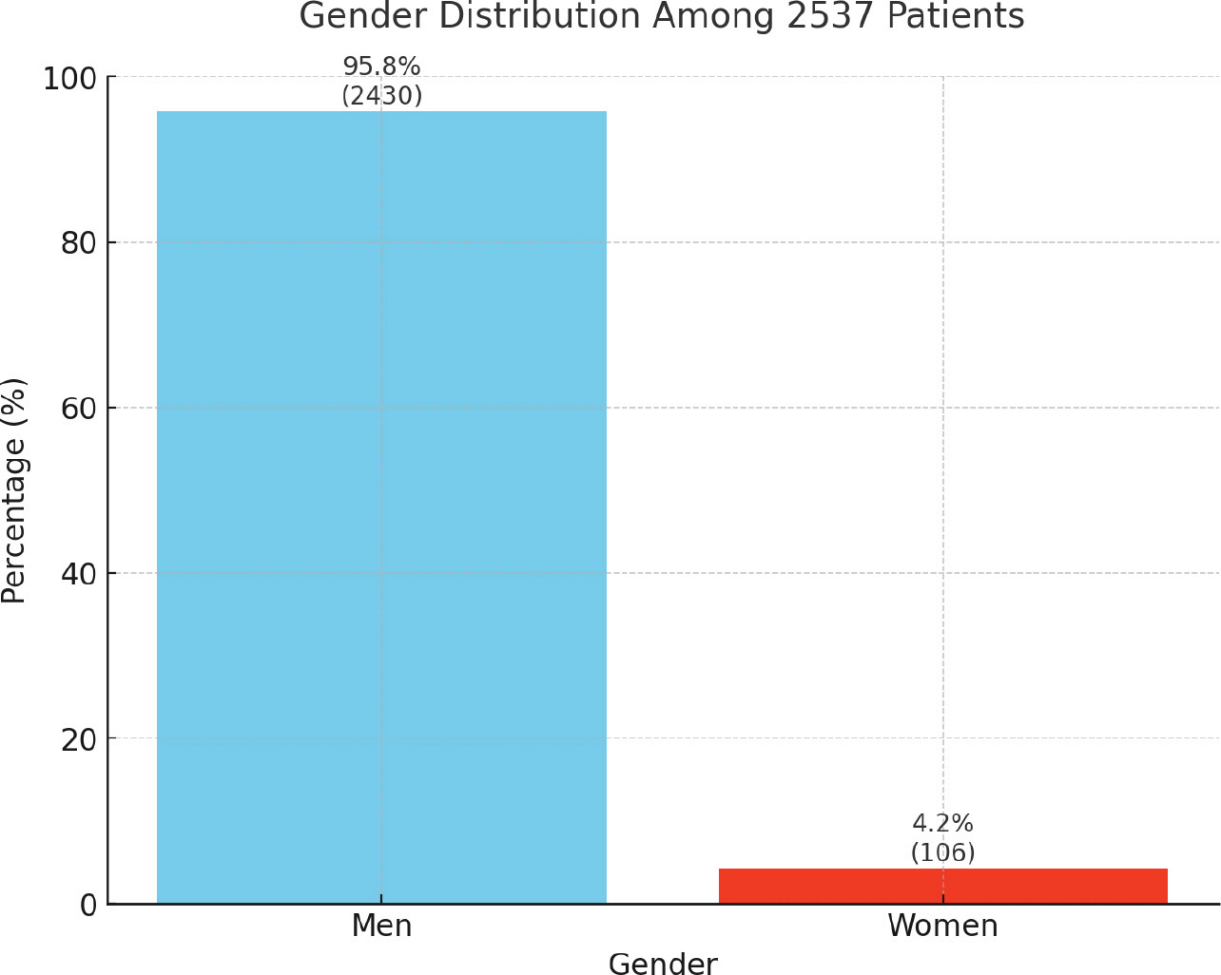
Gender distribution.

**Figure 3:**
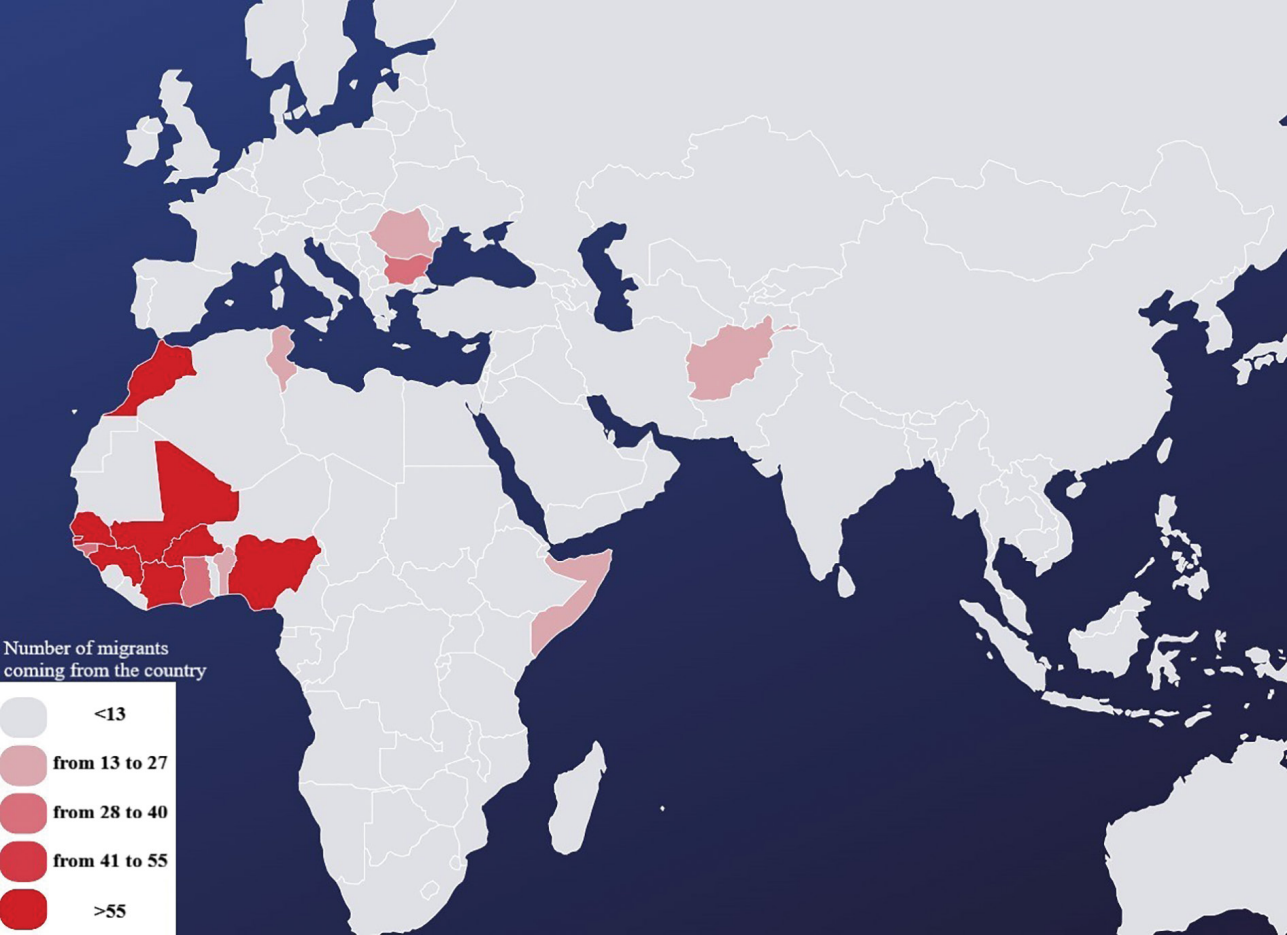
Countries of origins.

The analysis of 2,537 first visits revealed a significant gender imbalance, with male patients comprising 95.8% of the sample and female patients only 4.2% (Table 1; Figure 2). The gender distribution varied across settlements, with women representing 26.5% in Borgo Tressanti but only 0.4% in Arena and 0% in Turi. Settlement sizes ranged from 7 patients in San Carlo d’Ascoli (0.3%) to 843 in Pista (33.2%) and 650 in Casa Sankara (25.6%) (Table 1). Patient nationalities were varied, with Senegal (27.0%), Gambia (20.6%), Morocco (13.6%), Mali (10.0%), and Nigeria (5.4%) being the most represented (Table 2). Variations by settlement were notable: for instance, Senegalese migrants dominated in Casa Sankara, while Gambian patients were primarily concentrated in Pista. Figure 3 maps the geographic origins of the migrants.

**Table 1:** Absolute distribution and percentage of patients at the first visit by gender and location.

GENDER SETTLEMENT	MALE 2,430 (95.8%)	FEMALE 107 (4.2%)	TOTAL 2,537 (%)
Arena (A)	275 (99.6%)	1 (0.4%)	276 (10.9%)
Barletta (B)	15 (100%)	0 (0%)	15 (0.6%)
Borgo Tressanti (C)	50 (73.5%)	18 (26.5%)	68 (2.7%)
Contrada Cicerone (D)	167 (99.4%)	1 (0.6%)	168 (6.6%)
Madonna di Ripalta (E)	78 (95.1%)	4 (4.9%)	82 (3.2%)
Pista (F)	773 (91.7%)	70 (8.3%)	843 (33.2%)
San Carlo d’Ascoli (G)	7 (100%)	0 (0%)	7 (0.3%)
Sankara (H)	643 (98.9%)	7 (1.1%)	650 (25.6%)
Terlizzi (I)	63 (100%)	0 (0%)	63 (2.5%)
Terraneo (J)	90 (93.8%)	6 (6.3%)	96 (3.8%)
Turi (K)	223 (100%)	0 (0%)	223 (8.8%)
Uliveto (L)	46 (100%)	0 (0%)	46 (1.8%)

**Table 2:** Distribution of patient nationalities by settlement.

SETTLEMENT NATIONALITY	*N* (%)	A	B	C	D	E	F	G	H	I	J	K	L
Afghanistan	14 (0.55%)	0	0	0	0	0	14	0	0	0	0	0	0
Algeria	7 (0.28%)	0	1	0	0	0	0	0	0	0	0	6	0
Bangladesh	1 (0.04%)	0	0	0	0	0	1	0	0	0	0	0	0
Belgium	1 (0.04%)	0	0	0	0	0	0	0	1	0	0	0	0
Benin	17 (0.67%)	8	0	0	0	0	5	0	4	0	0	0	0
Bulgaria	29 (1.14%)	0	0	28	1	0	0	0	0	0	0	0	0
Burkina Faso	109 (4.3%)	1	0	0	2	62	8	2	3	0	31	0	0
Cameroon	8 (0.32%)	0	0	0	0	1	3	0	4	0	0	0	0
Chad	4 (0.16%)	1	0	0	1	0	2	0	0	0	0	0	0
Congo	3 (0.12%)	0	0	0	3	0	0	0	0	0	0	0	0
Ivory Coast	86 (3.39%)	16	0	0	28	4	20	0	16	0	2	0	0
Ethiopia	1 (0.04%)	0	0	0	0	0	1	0	0	0	0	0	0
Gambia	523 (20.61%)	101	0	0	0	0	168	0	251	0	2	1	0
Ghana	33 (1.3%)	2	0	0	1	0	21	0	2	0	7	0	0
Guinea‑Bissau	31 (1.22%)	4	0	0	1	0	16	0	10	0	0	0	0
Guinea‑Conakry	110 (4.34%)	11	0	0	13	1	55	0	30	0	0	0	0
India	5 (0.2%)	0	0	0	0	5	0	0	0	0	0	0	0
Italy	3 (0.12%)	0	0	1	0	0	0	0	0	0	2	0	0
Liberia	3 (0.12%)	0	0	0	0	1	2	0	0	0	0	0	0
Libya	1 (0.04%)	0	0	0	0	0	0	0	0	0	1	0	0
Mali	254 (10.01%)	42	0	0	103	4	54	1	44	0	5	1	0
Morocco	344 (13.56%)	0	2	26	0	0	2	1	1	55	6	209	42
Mauritania	3 (0.12%)	0	0	0	0	0	2	0	1	0	0	0	0
Mauritius	1 (0.04%)	0	0	0	0	0	0	0	1	0	0	0	0
Somalia (Mogadishu)	1 (0.04%)	0	0	0	0	0	1	0	0	0	0	0	0
Nigeria	137 (5.4%)	1	0	0	0	0	112	0	10	0	14	0	0
Pakistan	8 (0.32%)	0	0	0	0	0	8	0	0	0	0	0	0
Poland	9 (0.35%)	0	0	0	0	1	0	0	0	0	8	0	0
Punjab (India/Pakistan)	2 (0.08%)	0	0	0	0	2	0	0	0	0	0	0	0
Romania	24 (0.95%)	0	0	7	0	0	0	3	0	0	14	0	0
Senegal	686 (27.04%)	85	0	0	15	1	315	0	263	0	3	4	0
Sierra Leone	11 (0.43%)	2	0	0	0	0	5	0	4	0	0	0	0
Somalia	15 (0.59%)	0	0	0	0	0	15	0	0	0	0	0	0
Sudan	3 (0.12%)	0	0	0	0	0	3	0	0	0	0	0	0
Togo	3 (0.12%)	0	0	0	0	0	2	0	1	0	0	0	0
Tunisia	26 (1.02%)	0	12	5	0	0	0	0	0	6	0	2	1
Not available	21 (0.83%)	2	0	1	0	0	8	0	4	2	1	0	3

A = Arena; B = Barletta; C = Borgo Tressanti; D = Contrada Cicerone; E = Madonna di Ripalta; F = Pista; G = San Carlo d’Ascoli; H = Sankara; I = Terlizzi; J = Terraneo; K = Turi; L = Uliveto.

### Socio‑Health access

More than half of the patients (52.3%) were aged 30–45 years, followed by 19–29 years (30.9%) and 46–65 years (12.5%) (Table 3). Only 17.9% of patients had residence permits, and a mere 7.3% had access to a general practitioner. Access to healthcare also varied across settlements. Long‑term data collection (2017–2023) was available for Casa Sankara and Arena, while settlements such as Barletta and Uliveto contributed data only in recent years (Table 3).

**Table 3:** Distribution by settlement of age at first visit, presence of residence permit, presence of GP, and year of first visit.

AGE IN YEARS	TOTAL (%)	A	B	C	D	E	F	G	H	I	J	K	L
0–18	42 (1.66%)	1	0	8	0	6	4	0	3	1	9	1	9
19–29	783 (30.86%)	88	7	8	31	13	288	1	242	41	10	36	18
30–45	1,327 (52.31%)	169	6	34	118	31	454	3	345	14	38	103	12
46–65	317 (12.50%)	12	2	14	18	32	72	2	51	6	35	71	2
Over 65	19 (0.75%)	0	0	2	0	0	6	1	4	1	1	4	0
Not available	49 (1.93%)	6	0	2	1	0	19	0	5	0	3	8	5
Median	29.7	29.5	28.9	35.6	32.6	40.2	26.9	45.0	28.9	26.0	39.8	36.7	25.5
Average (SD)	31.8 (10.4)	30.7 (6.8)	31.1 (8.3)	35.6 (14.3)	34.00 (7.8)	39.2 (13.9)	28.9 (8.4)	49.5 (21.2)	30.9 (8.7)	29.7 (9.8)	39.1 (14.8)	39.5 (13.4)	29.1 (12.6)
**RESIDENCE PERMIT**	**TOTAL (%)**	**A**	**B**	**C**	**D**	**E**	**F**	**G**	**H**	**I**	**J**	**K**	**L**
Absent	2,083 (82.10%)	191	15	60	71	69	838	7	452	59	62	210	46
Present	454 (17.90%)	85	0	8	97	13	5	0	198	4	34	13	0
**GENERAL PRATICTIONER**	**TOTAL (%)**	**A**	**B**	**C**	**D**	**E**	**F**	**G**	**H**	**I**	**J**	**K**	**L**
Absent	2,352 (92.71%)	258	15	66	160	82	733	7	614	63	89	221	44
Present	185 (7.29%)	18	0	2	8	0	110	0	36	0	7	2	2
**YEAR OF FIRST VISIT**	**TOTAL (%)**	**A**	**B**	**C**	**D**	**E**	**F**	**G**	**H**	**I**	**J**	**K**	**L**
2017	97 (3.82%)	16	0	0	0	0	33	0	48	0	0	0	0
2018	574 (22.63%)	41	0	0	0	0	408	0	125	0	0	0	0
2019	447 (17.62%)	26	0	0	0	0	334	0	87	0	0	0	0
2020	465 (18.33%)	111	0	0	62	0	68	0	190	0	34	0	0
2021	243 (9.58%)	43	0	0	74	0	0	0	77	0	49	0	0
2022	338 (13.32%)	17	15	0	19	53	0	1	60	0	12	161	0
2023	373 (14.70%)	22	0	68	13	29	0	6	63	63	1	62	46

A = Arena; B = Barletta; C = Borgo Tressanti; D = Contrada Cicerone; E = Madonna di Ripalta; F = Pista; G = San Carlo d’Ascoli; H = Sankara; I = Terlizzi; J = Terraneo; K = Turi; L = Uliveto.

### Clinical conditions

At the first visit, fatigue‑related syndromes and musculoskeletal disorders were the most frequently diagnosed conditions, affecting 27.3% of patients, followed by gastrointestinal issues (14.7%), respiratory infections (14.3%), dermatological conditions (12.1%), and cardiovascular diseases (8.2%) (Table 6). Chronic conditions such as hypertension (3.5%), diabetes (0.9%), and asthma or chronic bronchitis (1.2%) were less common. Among infectious diseases, tuberculosis (TB) (0.9%) was the most prevalent, followed by hepatitis B virus (HBV) (0.4%), hepatitis C virus (0.1%), and HIV (0.2%) (Table 4). In total, seven cases of neoplasms were identified, including in one patient suffering from osteosarcoma of the right lower limb and in one patient suffering from sarcoma of the jaw. Both patients were followed up with by the CUAMM doctors during the therapeutic process.

**Table 4:** Distribution by location of clinical characteristics recorded at the first visit.

PATHOLOGY	TOTAL (%)	A	B	C	D	E	F	G	H	I	J	K	L
**Diabetes**													
Absent	2,514 (99.09%)	273	15	66	167	82	840	7	644	62	93	221	44
Present	23 (0.91%)	3	0	2	1	0	3	0	6	1	3	2	2
**High blood pressure**													
Absent	2,448 (96.49%)	269	15	65	158	79	831	5	620	63	83	215	45
Present	89 (3.51%)	7	0	3	10	3	12	2	30	0	13	8	1
**“Fatigue” pathologies**													
Absent	2,036 (80.25%)	229	15	56	134	64	624	5	531	52	84	206	36
Present	501 (19.75%)	47	0	12	34	18	219	2	119	11	12	17	10
**Asthma or chronic bronchitis**													
Absent	2,507 (98.82%)	273	15	67	168	82	836	7	636	63	96	220	44
Present	30 (1.18%)	3	0	1	0	0	7	0	14	0	0	3	2
**Tubercolosis**													
Absent	2,515 (99.13%)	274	15	68	168	82	837	7	637	63	96	222	46
Present	22 (0.87%)	2	0	0	0	0	6	0	13	0	0	1	0
**HIV**													
Absent	2,531 (99.76%)	273	15	68	167	81	843	7	649	63	96	223	46
Present	6 (0.24%)	3	0	0	1	1	0	0	1	0	0	0	0
**HBV**													
Absent	2,528 (99.65%)	273	15	68	168	82	841	7	646	63	96	223	46
Present	9 (0.35%)	3	0	0	0	0	2	0	4	0	0	0	0
**HCV**													
Absent	2,534 (99.88%)	275	15	68	168	82	843	7	648	63	96	223	46
Present	3 (0.12%)	1	0	0	0	0	0	0	2	0	0	0	0

A = Arena; B = Barletta; C = Borgo Tressanti; D = Contrada Cicerone; E = Madonna di Ripalta; F = Pista; G = San Carlo d’Ascoli; H = Sankara; I = Terlizzi; J = Terraneo; K = Turi; L = Uliveto.

### Visits

**Figure 4:**
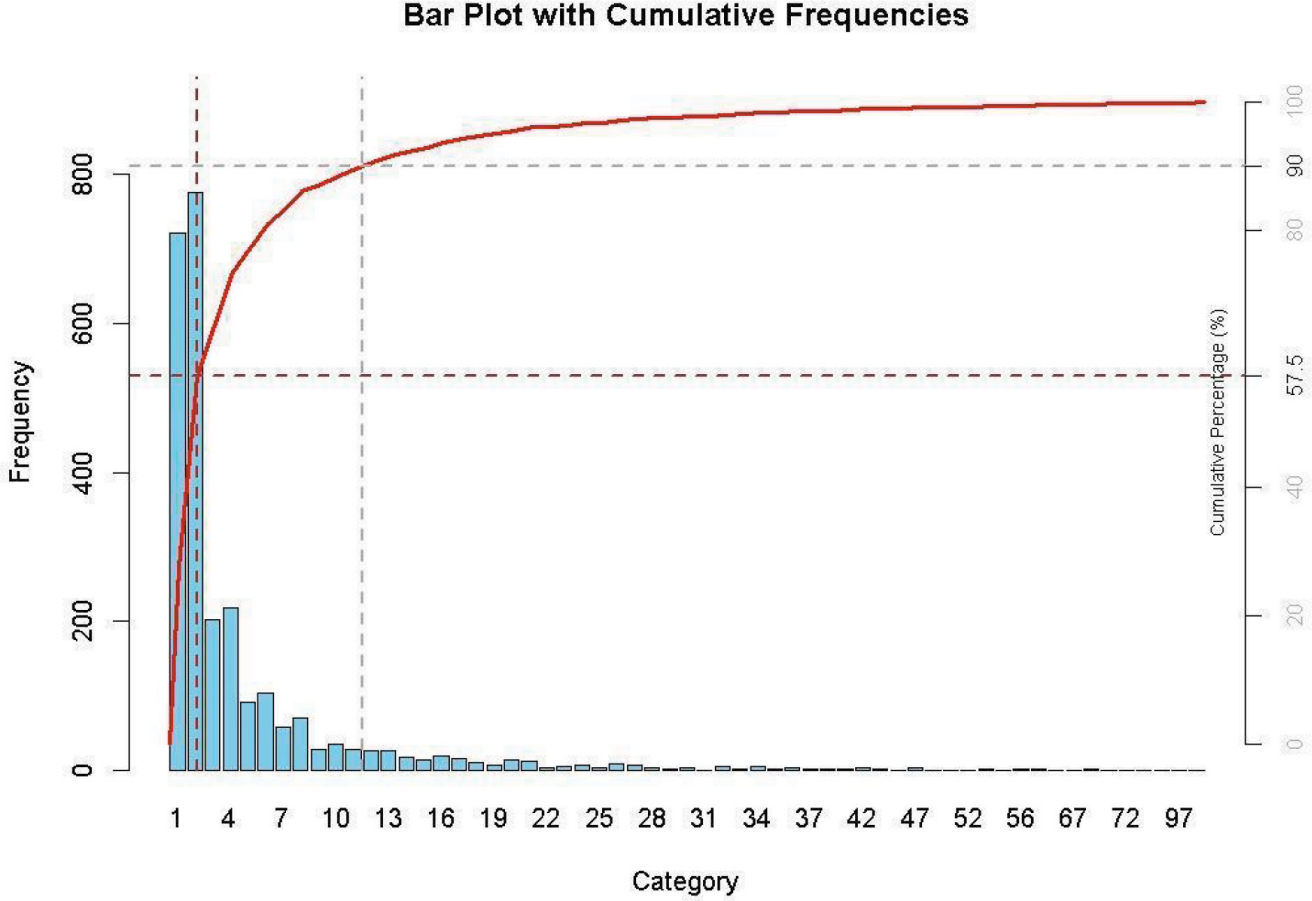
Pareto chart of total number of visits per patient.

Over the study period, a total of 13,103 visits were conducted, with male patients accounting for 97.8% and female patients 2.2% (Table 5). The average number of visits per patient was 5.16, with the highest averages recorded in Arena (9.6 visits per patient) and Casa Sankara (8.18 visits per patient). Figure 4 illustrates the frequency of visits per patient, showing that 90% attended a maximum of 11 visits, while 42.5% had more than 2 visits. Table 6 shows the distribution of pathologies by settlement.

**Table 5:** Distribution of the number of visits by settlement.

	MALES *N* (%) 12,809 (97.8%)	FEMALE *N* (%) 294 (2.2%)	TOTAL *N* (%) 13,103 (100%)	AVERAGE NUMBER OF VISITS PER PATIENT 5.16
Arena	2,647 (99.9%)	3 (0.1%)	2,650 (20.2%)	9.6
Barletta	40 (100%)	0 (0%)	40 (0.3%)	2.67
Borgo Tressanti	127 (66.1%)	65 (33.9%)	192 (1.5%)	2.82
Contrada Cicerone	1,062 (99.7%)	3 (0.3%)	1,065 (8.1%)	6.34
Madonna di Ripalta	410 (94%)	26 (6%)	436 (3.3%)	5.32
Pista	1,517 (91.1%)	148 (8.9%)	1,665 (12.7%)	1.98
San Carlo d’Ascoli	51 (100%)	0 (0%)	51 (0.4%)	7.29
Sankara	5,281 (99.4%)	34 (0.6%)	5,315 (40.6%)	8.18
Terlizzi	160 (100%)	0 (0%)	160 (1.2%)	2.54
Terraneo	785 (98.1%)	15 (1.9%)	800 (6.1%)	8.33
Turi	653 (100%)	0 (0%)	653 (5%)	2.93
Uliveto	76 (100%)	0 (0%)	76 (0.6%)	1.65

**Table 6:** Distribution of pathologies by settlement.

DIAGNOSIS *N* (%)	A 1,217 (17.9%)	B 18 (0.3%)	C 129 (1.9%)	D 461 (6.8%)	E 218 (3.2%)	F 1,170 (17.2%)	G 26 (0.4%)	H 2,710 (40%)	I 96 (1.4%)	J 331 (4.9%)	K 350 (5.2%)	L 57 (0.8%)	TOTAL 6,783 (100%)
Fatigue disorders (tiredness and osteoarticular disorders)	297 (24.4%)	5 (27.8%)	36 (27.9%)	125 (27.1%)	82 (37.6%)	334 (28.6%)	11 (42.3%)	775 (28.6%)	23 (23.9%)	66 (20.0%)	86 (24.6%)	16 (28.1%)	**1,856 (27.3%)**
Gastrointestinal disorders	180 (14.8%)	1 (5.6%)	11 (8.5%)	81 (17.6%)	23 (10.6%)	225 (19.2%)	0 (0%)	412 (15.2%)	4 (4.2%)	14 (4.2%)	47 (13.4%)	2 (3.5%)	**1,000 (14.7%)**
Airways pathologies	158 (13%)	2 (11.1%)	21 (16.3%)	52 (11.3%)	19 (8.7%)	207 (17.7%)	1 (3.8%)	421 (15.5%)	19 (19.8%)	21 (6.3%)	37 (10.6%)	9 (15.8%)	**967 (14.3%)**
Dermatological	174 (14.3%)	4 (22.2%)	8 (6.2%)	90 (19.5%)	22 (10.1%)	120 (10.3%)	1 (3.8%)	279 (10.3%)	25 (26%)	41 (12.4%)	46 (13.1%)	13 (22.8%)	**823 (12.1%)**
Cardiovascular	70 (5.8%)	2 (11.1%)	15 (11.6%)	44 (9.5%)	37 (17%)	31 (2.6%)	10 (38.5%)	201 (7.4%)	0 (0%)	119 (36%)	24 (6.9%)	2 (3.5%)	**555 (8.2%)**
Dental problems	109 (9%)	1 (5.6%)	7 (5.4%)	26 (5.6%)	5 (2.3%)	103 (8.8%)	0 (0%)	186 (6.9%)	8 (8.3%)	9 (2.7%)	32 (9.1%)	4 (7%)	**490 (7.2%)**
Traumas	91 (7.5%)	1 (5.6%)	2 (1.6%)	9 (2%)	5 (2.3%)	40 (3.4%)	2 (7.7%)	134 (4.9%)	5 (5.2%)	12 (3.6%)	17 (4.9%)	6 (10.5%)	**324 (4.8%)**
Ophthalmological	56 (4.6%)	1 (5.6%)	4 (3.1%)	21 (4.6%)	10 (4.6%)	35 (3%)	1 (3.8%)	100 (3.7%)	4 (4.2%)	25 (7.6%)	34 (9.7%)	2 (3.5%)	**293 (4.3%)**
Urinary tract	31 (2.5%)	0 (0%)	2 (1.6%)	3 (0.7%)	5 (2.3%)	31 (2.6%)	0 (0%)	31 (1.1%)	0 (0%)	4 (1.2%)	10 (2.9%)	0 (0%)	**117 (1.7%)**
ENT pathologies	20 (1.6%)	1 (5.6%)	2 (1.6%)	4 (0.9%)	5 (2.3%)	5 (0.4%)	0 (0%)	42 (1.5%)	1 (1%)	0 (0%)	6 (1.7%)	0 (0%)	**86 (1.3%)**
Diabetes and endocrinological disorders	4 (0.3%)	0 (0%)	14 (10.9%)	4 (0.9%)	1 (0.5%)	4 (0.3%)	0 (0%)	23 (0.8%)	3 (3,1%)	18 (5.4%)	5 (1.4%)	2 (3.5%)	**78 (1.2%)**
Neuropsychiatric disorders	16 (1%)	0 (0%)	5 (3.9%)	1 (0.2%)	1 (0.5%)	5 (0.5%)	0 (0%)	31 (1.1%)	3 (3.1%)	2 (0.6%)	5 (1.4%)	1 (1.8%)	**70 (1.1%)**
TB	6 (0.5%)	0 (0%)	0 (0%)	0 (0%)	0 (0%)	6 (0.5%)	0 (0%)	26 (1%)	0 (0%)	0 (0%)	1 (0.3%)	0 (0%)	**39 (0.6%)**
Rheumatological	2 (0.2%)	0 (0%)	0 (0%)	0 (0%)	0 (0%)	1 (0.1%)	0 (0%)	35 (1.3%)	0 (0%)	0 (0%)	0 (0%)	0 (0%)	**38 (0.6%)**
Obstetric‑gynecological	0 (0%)	0 (0%)	1 (0.8%)	0 (0%)	0 (0%)	23 (2%)	0 (0%)	0 (0%)	0 (0%)	0 (0%)	0 (0%)	0 (0%)	**24 (0.4%)**
HIV	3 (0.2%)	0 (0%)	0 (0%)	1 (0.2%)	3 (1.4%)	0 (0%)	0 (0%)	2 (0.1%)	0 (0%)	0 (0%)	0 (0%)	0 (0%)	**9 (0.1%)**
Oncological	0 (0%)	0 (0%)	1 (0.8%)	0 (0%)	0 (0%)	0 (0%)	0 (0%)	5 (0.2%)	1 (1%)	0 (0%)	0 (0%)	0 (0%)	**7 (0.1%)**
Hematological	0 (0%)	0 (0%)	0 (0%)	0 (0%)	0 (0%)	0 (0%)	0 (0%)	7 (0.3%)	0 (0%)	0 (0%)	0 (0%)	0 (0%)	**7 (0.1%)**

A = Arena; B = Barletta; C = Borgo Tressanti; D = Contrada Cicerone; E = Madonna di Ripalta; F = Pista; G = San Carlo d’Ascoli; H = Sankara; I = Terlizzi; J = Terraneo; K = Turi; L = Uliveto; ENT = ears, nose, and throat.

### Treatments

The most frequently prescribed therapies included anti‑inflammatory drugs (33.6%), topical treatments (11.5%), supplements (10.7%), and gastroprotective agents (6.8%) (Table 7). Treatments varied by condition. For fatigue‑related syndromes, 76% of patients received anti‑inflammatory drugs. Gastroprotective agents were the primary treatment for gastrointestinal disorders (52%), while dermatological conditions were primarily addressed with topical treatments (57%).

**Table 7:** Therapies administered.

THERAPIES *N* (%)	A 1,217 (17.9%)	B 18 (0.3%)	C 129 (1.9%)	D 461 (6.8%)	E 218 (3.2%)	F 1,170 (17.2%)	G 26 (0.4%)	H 2,710 (40%)	I 96 (1.4%)	J 331 (4.9%)	K 350 (5.2%)	L 57 (0.8%)	TOTAL 6,783 (100%)
NSAIDs, painkillers and cortisone	394 (30.9%)	5 (27.8%)	29 (22.5%)	132 (28.7%)	66 (30.3%)	478 (40.9%)	10 (38.4%)	951 (35.1%)	33 (34.3%)	70 (21.1%)	97 (27.7%)	7 (12.3%)	**2272 (33.6%)**
Topicals (creams and eye drops)	159 (13.1%)	2 (11.1%)	7 (5.4%)	70 (15.2%)	34 (15.6%)	122 (10.4%)	0 (0%)	296 (10.9%)	17 (17.7%)	42 (12.7%)	21 (6%)	12 (21.1%)	**782 (11.5%)**
Adjuvants, supplements, and probiotics	122 (10%)	3 (16.7%)	13 (10.1%)	62 (13.4%)	30 (13.8%)	103 (8.8%)	2 (7.7%)	300 (11.1%)	7 (7.3%)	30 (9.1%)	47 (13.4%)	7 (12.3%)	**726 (10.7%)**
Gastrointestinal	99 (8.1%)	1 (5.6%)	6 (4.7%)	44 (9.5%)	12 (5.6%)	137 (11.7%)	0 (0%)	229 (8.4%)	3 (3.1%)	6 (1.8%)	30 (8.5%)	1 (1.8%)	**568 (8.3%)**
Antibiotics, antifungals, and antiparasitics	102 (8.4%)	0 (0%)	13 (10.1%)	25 (5.4%)	5 (2.3%)	136 (11.6%)	1 (3.8%)	189 (7.0%)	18 (18.8%)	10 (3.0%)	22 (6.3%)	8 (14.0%)	**529 (7.9%)**
Cardiovascular (antihypertensives, diuretics, and anticoagulants)	56 (4.5%)	0 (0%)	10 (7.8%)	28 (6.1%)	27 (12.5%)	30 (2.6%)	10 (38.5%)	161 (5.9%)	0 (0%)	102 (30.8%)	18 (5.2%)	1 (1.8%)	**443 (6.5%)**
Respiratory tract treatments	75 (6.1%)	2 (11.1%)	11 (8.6%)	15 (3.2%)	1 (0.5%)	80 (6.8%)	1 (3.8%)	193 (7.1%)	5 (5.2%)	13 (3.9%)	28 (8.0%)	4 (7.1%)	**428 (6.3%)**
Medication	38 (3.1%)	0 (0%)	1 (0.8%)	14 (3%)	1 (0.5%)	14 (1.2%)	2 (7.7%)	61 (2.3%)	1 (1%)	8 (2.4%)	13 (3.7%)	5 (8.8%)	**158 (2.3%)**
Antidiabetics	1 (0.1%)	0 (0%)	12 (9.4%)	1 (0.2%)	1 (0.5%)	2 (0.2%)	0 (0%)	17 (0.7%)	2 (2.0%)	15 (4.5%)	3 (0.9%)	2 (3.6%)	**56 (1.0%)**
Neurological	0 (0%)	0 (0%)	1 (0.8%)	1 (0.2%)	0 (0%)	3 (0.3%)	0 (0%)	3 (0.1%)	1 (1%)	0 (0%)	2 (0.6%)	0 (0%)	**11 (0.2%)**
Health orientation and hospital care	3 (0.3%)	0 (0%)	0 (0%)	1 (0.2%)	0 (0%)	1 (0.1%)	0 (0%)	11 (0.4%)	0 (0%)	1 (0.3%)	1 (0.3%)	0 (0%)	**18 (0.2%)**
Not available	168 (13.8%)	5 (27.8%)	26 (20.2%)	68 (14.8%)	41 (18.8%)	64 (5.5%)	0 (0%)	299 (11%)	9 (9.4%)	34 (10.3%)	68 (19.4%)	10 (17.5%)	**792 (11.7%)**

A = Arena; B = Barletta; C = Borgo Tressanti; D = Contrada Cicerone; E = Madonna di Ripalta; F = Pista; G = San Carlo d’Ascoli; H = Sankara; I = Terlizzi; J = Terraneo; K = Turi; L = Uliveto; NSAIDs = nonsteroidal anti‑inflammatory drugs.

### Follow‑Up visits

The likelihood of follow‑up visits, considered a proxy for trust in the socio‑healthcare services provided by volunteers of Doctors with Africa CUAMM, was analyzed using logistic regression (Table 8). Compared with the reference settlement, Casa Sankara, which had the highest total number of visits, the settlements Arena, Contrada Cicerone, and Terraneo showed significantly higher odds of follow‑up visits. Specifically, the increases were +22% (OR = 2.22; 95% CI: 1.12–1.34; *p* < 0.001), +36% (OR = 1.36; 95% CI: 1.19–1.56; *p* < 0.001), and +47% (OR = 1.47; 95% CI: 1.27–1.71; *p* < 0.001), respectively.

**Table 8:** Simple logistic regression (unadjusted OR) and multiple logistic regression (adjusted OR) on the probability of returning to the visit with respect to the reference settlement Casa Sankara.

	UNADJUSTED ORS			ADJUSTED OR		
	OR	95% CI	*P*‑VALUE	OR	95% CI	*P*‑VALUE
**Settlements**						
Sankara (H)	ref.	–	–	ref.	–	–
Arena (A)	**1.22**	**1.12–1.34**	**<0.001**	**1.16**	**1.06–1.28**	**0.002**
Barletta (B)	1.27	0.68–2.38	0.451	**1.92**	**1.01–3.65**	**0.045**
Borgo Tressanti (C)	**0.51**	**0.37–0.69**	**0.018**	0.70	0.49–1.01	0.055
Contrada Cicerone (D)	**1.36**	**1.19–1.56**	**<0.001**	**1.34**	**1.18–1.54**	**<0.001**
Madonna di Ripalta (E)	1.04	0.86–1.26	0.692	**1.64**	**1.29–2.09**	**0.001**
Pista (F)	**0.45**	**0.39–0.50**	**<0.001**	**0.40**	**0.36–0.46**	**<0.001**
San Carlo d’Ascoli (G)	1.00	0.58–1.74	0.999	0.96	0.50–1.82	0.897
Terlizzi (I)	**0.69**	**0.50–0.96**	**0.025**	1.02	0.71–1.45	0.932
Terraneo (J)	**1.47**	**1.27–1.71**	**<0.001**	**1.39**	**1.18–1.64**	**0.001**
Turi (K)	0.90	0.77–1.06	0.208	**1.41**	**1.14–1.76**	**0.002**
Uliveto (L)	**0.35**	**0.21–0.58**	**<0.001**	**0.52**	**0.29–0.93**	**0.027**
**Gender**						
Male	ref.	–	–	ref.	–	–
Female	**0.71**	**0.56–0.90**	**0.005**	1.10	0.83–1.44	0.514
**Age range at first visit (years)**						
0–18	ref.	–	–	ref.	–	–
19–29	0.86	0.60–1.23	0.401	1.00	0.67–1.50	0.987
30–45	0.81	0.57–1.17	0.264	0.93	0.62–1.39	0.729
46–64	0.84	0.58–1.21	0.345	0.87	0.58–1.30	0.492
65+	0.61	0.34–1.08	0.087	0.92	0.50–1.71	0.804
**Country of origin**						
Other nations	ref.	–	–	ref.	–	–
Romania	**2.88**	**1.80–4.6**	**<0.001**	**3.09**	**1.79–5.34**	**<0.001**
**Year of first visit**						
<2022	ref.	–	–	ref.	–	–
2022–2023	**0.76**	**0.70–0.83**	**<0.001**	**0.60**	**0.52–0.70**	**<0.001**
**Diagnosis of TB**						
No	ref.	–	–	ref.	–	–
Yes	**0.63**	**0.49–0.81**	**<0.001**	**0.63**	**0.49–0.81**	**<0.001**

CI = confidence interval; OR = odds ratio

In contrast, settlements such as Borgo Tressanti, Pista, Terlizzi, and Uliveto had significantly lower odds of follow‑up visits compared with Casa Sankara. The decreases were −49% (OR = 0.51; 95% CI: 0.37–0.69; *p* = 0.018), −55% (OR = 0.45; 95% CI: 0.39–0.50; *p* < 0.001), −31% (OR = 0.69; 95% CI: 0.50–0.96; *p* = 0.025), and −65% (OR = 0.35; 95% CI: 0.21–0.58; *p* < 0.001), respectively.

When additional variables were controlled for in the multiple logistic regression model, statistical significance was maintained for Arena (+16%; OR = 1.16; *p* = 0.002), Contrada Cicerone (+34%; OR = 1.34; *p* < 0.001), Terraneo (+39%; OR = 1.39; *p* = 0.001), and Uliveto (− 48%; OR = 0.52; *p* = 0.027). However, settlements such as Borgo Tressanti (*p* = 0.055) and Terlizzi (*p* = 0.932) lost statistical significance, suggesting the influence of confounding factors.

Further analysis revealed that settlements such as Barletta (OR = 1.92; 95% CI: 1.01–3.65; *p* = 0.045), Madonna di Ripalta (OR = 1.64; 95% CI: 1.29–2.09; *p* = 0.001), and Turi (OR = 1.41; 95% CI: 1.14–1.76; *p* = 0.002) had significantly higher odds of follow‑up visits compared with Casa Sankara. Regarding gender, female patients were significantly less likely to attend follow‑up visits compared with male patients (*p* = 0.005).

For a better understanding, see Figure 1, where you will find a map of the informal settlements covered by the analysis. The most widespread nationality of migrants living in each settlement is indicated.

## Discussion

This study aims to highlight the health needs and social conditions of migrant agricultural workers in Puglia’s informal settlements, often overlooked by institutions. By analyzing 13,103 visits from 2,537 patients, the research emphasizes critical health and socio‑health issues, offering valuable insights for targeted interventions. Addressing these needs is not only a healthcare matter but also a step toward social inclusion and justice. The predominance of male patients (95.8%) reflects both demographic trends and cultural barriers limiting women’s healthcare access. Migrant men dominate sectors such as agriculture, construction, and logistics, while women are typically employed in domestic or care work in urban areas. The central Mediterranean migration route, one of the most dangerous, discourages families from sending women owing to the higher risks of violence and exploitation [10]. Even when women seek healthcare, their follow‑up rates are lower, possibly owing to discomfort, fear of judgment, or the prioritization of family needs over their own health.

Most patients (85%) come from sub‑Saharan African countries such as Senegal, Gambia, and Mali, regions marked by poverty, instability, and limited opportunities that drive migration through dangerous routes such as Libya and the Mediterranean [10]. Only 18% of patients had residence permits, often owing to exploitation practices such as document confiscation by labor intermediaries (*caporali*). This perpetuates exclusion from healthcare, as fear of deportation discourages migrants from seeking medical help [11, 12].

Despite Italy’s universal healthcare system, only 7% of patients had a general practitioner. This is due to low awareness of healthcare rights and difficulties in obtaining the Temporarily Present Foreigner (STP) Code, which is necessary for accessing basic services. Migrants’ limited health literacy and marginalization further exacerbate this gap [13, 14].

The study highlights the burden of work‑related health issues. Fatigue syndromes and musculoskeletal pain made up over 27% of visits, reflecting harsh working conditions rather than age‑related issues. Injuries and trauma (5% of visits) align with literature on workplace hazards faced by migrants [6, 15, 16]. Poor hygiene and overcrowding contributed to frequent respiratory infections (14.3%), dermatological issues (12.1%), and dental problems (7.2%). Additionally, indoor use of charcoal and waste‑burning increased respiratory risks.

Infectious diseases such as hepatitis B and C, tuberculosis, and HIV were found at low rates, consistent with existing data on migrant populations [17–19]. However, given the high‑risk conditions in informal settlements and the limited availability of diagnostic resources, underreporting cannot be excluded. Previous CUAMM studies and external sources have reported higher prevalence rates in similar contexts, suggesting that systematic screening efforts might reveal a greater disease burden than observed in our dataset [20–22].

Periodic checks made it possible to diagnose seven cases of neoplasms, which would have come to the attention of public health workers at a late stage. The young age of migrants helps to explain the low prevalence of chronic diseases such as hypertension and diabetes.

The high prescription rates of anti‑inflammatory drugs (33.6%) reflect work‑related conditions, while supplements and gastroprotective agents address issues tied to poor nutrition, stress, and heavy nonsteroidal anti‑inflammatory drug (NSAID) use. Despite limited resources, the high follow‑up rate (average of 5.6 visits per patient) shows trust in CUAMM’s mobile clinics, especially in settlements such as Arena and Casa Sankara, where collaboration with local communities boosted engagement [23]. One key finding is the need to incorporate health and migration training in medical education. As the World Health Organization (WHO) recommends, healthcare professionals should be equipped to address the complex needs of migrant populations, including cultural dynamics and language barriers and building trust with vulnerable groups [24]. Without this training, providers risk perpetuating gaps in access and care quality for migrants [24–26]. Another fundamental objective would be to guarantee that migrants are taken into care even after their stay in the ghettos has ended, when they are no longer intercepted by CUAMM doctors and cultural mediators.

Study limitations include the heterogeneity of settlements and reliance on clinical diagnoses, which may have missed conditions requiring advanced diagnostics, such as diabetes, tuberculosis, and cancers. Furthermore, it was not possible to carry out systematic mental health screening, despite the literature [27, 28] indicating that migrants suffer more often from mental disorders than the host population. The lack of structured mental health data represents a gap that will be addressed in future healthcare interventions. Specifically, upcoming medical visits will integrate a targeted mental health assessment program to better evaluate psychological distress and psychiatric conditions among migrant workers.

## Conclusion

This study offers a comprehensive look at the health needs of migrant workers in Puglia, highlighting work‑ and living‑related health issues and barriers to accessing healthcare. It emphasizes the crucial role of CUAMM’s mobile clinics, which have become essential for isolated populations, offering primary care, referrals, and socio‑health support. To improve healthcare access, our experience suggests:

- Equitable access to healthcare: Implement information campaigns on healthcare rights and ensure the assignment of general practitioners. Address barriers such as low health literacy and language differences, which disproportionately affect migrants [13, 14, 29].
- Cultural competence: Train healthcare staff to address migrants’ needs, provide interpreters and cultural mediators, and develop protocols for more effective management of migrant health. Studies show that these measures significantly improve health outcomes [30–34].
- Incorporating health and migration training into medical education: Healthcare curricula should include modules on health and migration, as recommended by the WHO [24]. This training will enable healthcare providers to understand the unique needs of migrant populations and reduce systemic barriers to care.
- Integrated approach with institutions and partners: Strengthen collaboration between central (e.g., regional) and local institutions, including health services and administrative bodies, for a coordinated, sustainable response. Partnerships with NGOs are crucial to align actions with regional programming and offer comprehensive support to vulnerable populations. Coordinating resources and expertise can enhance the effectiveness of interventions.
- Role of universities: Universities play a key role in addressing health challenges through operational research and education. Research should provide evidence‑based solutions for migrant health, while universities also equip future medical professionals with the necessary skills to manage migration‑related health issues. This dual role is vital for long‑term improvements in healthcare systems and social equity.

In conclusion, mobile clinics have proven effective in bridging gaps in healthcare access for marginalized populations, reducing logistical barriers, and creating a trusted, welcoming environment. This model should be integrated into traditional healthcare systems to extend its reach and sustainability. Finally, addressing labor exploitation is essential for improving migrant health. Better working conditions and housing would reduce health risks and promote social inclusion, facilitating continuous care access. Socio‑healthcare services must be truly integrated, fostering equality and justice for all, leaving no one behind.
